# Supplementary material for: Interventions for Preventing Residential Fires in Vulnerable Neighbourhoods and Indigenous Communities: A Systematic Review of the Literature
Source: Int J Environ Res Public Health. 2022 Apr 29;19(9):5434. doi: 10.3390/ijerph19095434 (PMC9100970; doi:10.3390/ijerph19095434)
Supplement: Supplementary file 1 [file ijerph-19-05434-s001.zip › ijerph-1635025-supplementary.pdf]

Supplementary Report S1

Residential Fires Systematic Review Search Report
Search Results

Database/ Source	Date Searched	# of Results
MEDLINE (Ovid)	August 4, 2021	700
Embase (Ovid)	August 4, 2021	892
CENTRAL (Ovid)	August 4, 2021	94
Web of Science Core Collection	August 4, 2021	1511
Trophy	August 4, 2021	2
PAIS Index (ProQuest)	August 4, 2021	61
ERIC (EBSCO)	August 4, 2021	123
FireDOC	August 4, 2021	86
IEEE Xplore	August 4, 2021	23
Native Health Database	August 4, 2021	15
Google Scholar	August 4, 2021	221
Database search total	---	3,727
ProQuest Dissertations & Theses Global	August 5, 2021	22
The Networked Digital library of Theses and Dissertations (NDLTD)	August 5, 2021	57
PapersFirst (via WorldCat FirstSearch)	August 5, 2021	13
Proceedings (via WorldCat FirstSearch)	August 5, 2021	105
OpenGrey	August 5, 2021	2
Grey Literature Report	August 5, 2021	0

DesLibris	August 6, 2021	26
Australian Institute of Health and Welfare (https://www.aihw.gov.au/reports-data/)		0
New Zealand Ministry of Health Library (https://www.health.govt.nz/about-ministry/ministry-health-library)		1
Govinfo (https://www.govinfo.gov/)		0
Training Programs in Epidemiology and Public Health Interventions Network (TEPHINET)		0
Google Search targeted to organizations in Australia, New Zealand, US and Canada		394
**Total Grey Literature**	---	620

Search Strategies
MEDLINE

Link:
Click to run search
The above Jumpstart will only work for users who have access to this specific database.

Database:
Ovid MEDLINE(R) and Epub Ahead of Print, In-Process, In-Data-Review & Other Non-Indexed Citations, Daily and Versions(R) <1946 to August 03, 2021>

#	Query	Results from 4 Aug 2021
1	Fires/	9,812

2	housing/ or housing for the elderly/ or public housing/ or refugee camps/ or independent living/	29,509
3	Accidents, Home/	4,595
4	2 or 3	33,958
5	1 and 4	361
6	(fire? adj10 (hous* or home? or residen*)).tw,kw.	1,091
7	5 or 6	1,305
8	Accident prevention/	9,224
9	Health Promotion/	77,129
10	Health education/	62,060
11	pc.fs.	1,355,349
12	(prevent* or reduc* or decrease? or intervention? or promot* or improve? or program* or educat*).tw,kf.	9,526,161
13	or/8-12	10,125,853
14	7 and 13	800
15	limit 14 to yr="1990 -Current"	700

Embase

Link:
Click to run search
The above Jumpstart will only work for users who have access to this specific database.

Database:
Embase <1974 to 2021 August 03>

#	Query	Results from 4 Aug 2021
1	fire/	12,671
2	housing/	26,468
3	home for the aged/	11,195
4	refugee camp/	834
5	independent living/	5,509
6	home accident/	3,141
7	or/2-6	46,586
8	1 and 7	294
9	(fire? adj10 (hous* or home? or residen*)).tw,kw.	1,454
10	8 or 9	1,625
11	accident prevention/	15,837

12	health promotion/ or health education/	190,148
13	public health campaign/ or public health message/	3,443
14	pc.fs.	1,168,364
15	(prevent* or reduc* or decrease? or intervention? or promot* or improve? or program* or educat*).tw,kw.	12,257,839
16	or/11-15	12,810,115
17	10 and 16	999
18	limit 17 to yr="1990 -Current"	892

CENTRAL

Link:
Click to run search
The above Jumpstart will only work for users who have access to this specific database.

Database:
EBM Reviews - Cochrane Central Register of Controlled Trials <June 2021>

#	Query	Results from 4 Aug 2021
1	Fires/	87

2	housing/ or housing for the elderly/ or public housing/ or refugee camps/ or independent living/	950
3	Accidents, Home/	74
4	2 or 3	1,019
5	1 and 4	16
6	(fire? adj10 (hous* or home? or residen*)).tw,kw.	94
7	5 or 6	100
8	Accident prevention/	121
9	Health Promotion/	6,061
10	Health education/	4,033
11	pc.fs.	94,473
12	(prevent* or reduc* or decrease? or intervention? or promot* or improve? or program* or educat*).tw,kw.	1,083,734
13	or/8-12	1,102,459
14	7 and 13	97
15	limit 14 to yr="1990 -Current"	94

Web of Science Core Collection

4	#1 AND #2	limit to 1990-2021	1,511
3	#1 AND #2		1,516
2	TS=(prevent* or reduc* or decrease$ or intervention$ or promot* or improve$ or program* or educat*)		17,035,187
1	TS=((fire$ NEAR/10 hous*) OR (fire$ NEAR/10 home$) OR (fire$ NEAR/10 residen*))		3,223

TRoPHI
Freetext (All but Authors): "residential fire*"

PAIS Index
((MAINSUBJECT.EXACT("Fire") AND MAINSUBJECT.EXACT.EXPLODE("Housing")) OR AB(fire NEAR/10 hous*) OR AB(fire NEAR/10 home?) OR AB(fire? NEAR/10 residen*)) AND (MAINSUBJECT.EXACT("Fire Prevention") OR AB(prevent* OR reduc* OR decrease OR intervention OR promot* OR improve? OR program* OR educat*))

Additional limits - Date: After January 01 1990

ERIC

#	Query	Limiters/Expanders	Last Run Via	Results
S7	S1 AND S5	Limiters - Published Date: 19000101-20201231<br>Search modes - Boolean/Phrase	Interface - EBSCOhost Research Databases<br>Search Screen	123

			- Advanced Search<br><br>Database<br><br>- ERIC	
S6	S1 AND S5	Search modes<br><br>- Boolean/Phrase	Interface<br><br>- EBSCOhost Research Databases<br><br>Search Screen<br><br>- Advanced Search	125

			Database<br><br>- ERIC	
S5	S2 OR S3 OR S4	Search modes<br><br>- Boolean/Phrase	Interface<br><br>- EBSCOhost Research Databases<br><br>Search Screen<br><br>- Advanced Search	1,05 1,771

			Database<br>- ERIC	
S4	TI ((prevent* or reduc* or decrease# or intervention# or promot* or improve# or program* or educat*)) OR AB ((prevent* or reduc* or decrease# or intervention# or promot* or improve# or program* or educat*))	Search modes<br>- Boolean/Phrase	Interface<br>- EBSCOhost Research Databases<br>Search Screen<br>- Advanced Search<br>Database	1,049,084

			- ERIC	
S3	(DE "Health Promotion") OR (DE "Health Education")	Search modes - Boolean/Phrase	Interface - EBSCOhost Research Databases<br>Search Screen - Advanced Search<br>Database	18,919

			- ERIC	
S2	DE "Fire Protection"	Search modes - Boolean/Phrase	Interface - EBSCOhost Research Databases<br>Search Screen - Advanced Search<br>Database	697

			- ERIC	
S1	TI ((fire$ N10 hous*) OR (fire$ N10 home#) OR (fire$ N10 residen*)) OR AB ((fire$ N10 hous*) OR (fire$ N10 home#) OR (fire$ N10 residen*))	Search modes - Boolean/Phrase	Interface - EBSCOhost Research Databases<br>Search Screen - Advanced Search<br>Database	158

			- ERIC	

FireDOC
Title: residential fire AND publication date: 1990-2021

IEEE Xplore
("All Metadata":"residential fire" OR "All Metadata":"residential fires") OR ("All Metadata":"house fire" OR "All Metadata":"house fires") OR ("All Metadata":"home fire" OR "All Metadata":"home fires")
Filters Applied: 1990 - 2022

Native Health Database
Keywords: residential fire OR house fire OR home fire
Range Start: 1990
Range End: 2021
Google Scholar
"Residential|house|home+fire+prevention|intervention"
Publication date: 1990-2021

ProQuest Dissertations & Theses Global
(TI(residential NEAR/10 fire*) OR TI(hous* NEAR/10 fire*) OR TI(home NEAR/10 fire*)) AND (NOFT(prevent* OR reduc* OR decrease* OR intervention* OR promot* OR improve* OR program* OR educat*))
Additional limits - Date: From January 01 1990 to December 31 2021

Networked Digital library of Theses and Dissertations (NDLTD)
title:"residential fire" OR title:"residential fires"

PapersFirst (via WorldCat FirstSearch)
(kw: residential w10 fire*) OR (kw: hous* w10 fire*) OR (kw: home w10 fire*) AND (kw: prevent* OR kw: reduc* OR kw: decrease* OR kw: intervention* OR kw: promot* OR kw: improve* OR kw: program* OR kw: educat*) and yr: 1990-2021

Proceedings (via WorldCat FirstSearch)
(kw: residential w10 fire*) OR (kw: hous* w10 fire*) OR (kw: home w10 fire*) AND (kw: prevent* OR kw: reduc* OR kw: decrease* OR kw: intervention* OR kw: promot* OR kw: improve* OR kw: program* OR kw: educat*) and yr: 1990-2021

OpenGrey
"residential fire" OR "residential fires"

Grey Literature Report
"residential fire" OR "residential fires"

DesLibris
Residential fire
Limit to public documents

Australian Institute of Health and Welfare
“Residential fire”
“Residential fires”

New Zealand Ministry of Health Library
Residential fire

Govinfo
collection:(GPO OR BUDGET OR CZIC OR CFR OR CPD OR BILLS OR CCAL OR CPRT OR CDIR OR CDOC OR CHRG OR CREC OR CRECB OR CRI OR CRPT OR SERIALSET OR ECONI OR ERP OR ERIC OR FR OR GAOREPORTS OR HOB OR HMAN OR HJOURNAL OR LSA OR GOVPUB OR PAI OR PPP OR PLAW OR SMAN OR COMPS OR STATUTE OR USCODE OR USCOURTS OR GOVMAN) AND null AND title:(residential fire)
Training Programs in Epidemiology and Public Health Interventions Network (TEPHINET)
Residential fire
Google Search
residential fire+intervention|program|educationfiletype:pdf

Narrowed to each geographical area identified

First 100 results taken for each when >100 results available
